# Allergic Patients with Long-Term Asthma Display Low Levels of *Bifidobacterium adolescentis*

**DOI:** 10.1371/journal.pone.0147809

**Published:** 2016-02-03

**Authors:** Arancha Hevia, Christian Milani, Patricia López, Carmen D. Donado, Adriana Cuervo, Sonia González, Ana Suárez, Francesca Turroni, Miguel Gueimonde, Marco Ventura, Borja Sánchez, Abelardo Margolles

**Affiliations:** 1 Instituto de Productos Lácteos de Asturias (IPLA), Consejo Superior de Investigaciones Científicas (CSIC), Villaviciosa, Asturias, Spain; 2 Laboratory of Probiogenomics, Department of Life Sciences, University of Parma, Parma, Italy; 3 Immunology Area, Department of Functional Biology, University of Oviedo, Oviedo, Asturias, Spain; 4 Allergy Unit, Hospital Universitario Central de Asturias, Oviedo, Asturias, Spain; 5 Physiology Area, Department of Functional Biology, University of Oviedo, Oviedo, Asturias, Spain; University of Ulm, GERMANY

## Abstract

Accumulated evidence suggests a relationship between specific allergic processes, such as atopic eczema in children, and an aberrant fecal microbiota. However, little is known about the complete microbiota profile of adult individuals suffering from asthma. We determined the fecal microbiota in 21 adult patients suffering allergic asthma (age 39.43 ± 10.98 years old) and compare it with the fecal microbiota of 22 healthy controls (age 39.29 ± 9.21 years old) using culture independent techniques. An Ion-Torrent 16S rRNA gene-based amplification and sequencing protocol was used to determine the fecal microbiota profile of the individuals. Sequence microbiota analysis showed that the microbial alpha-diversity was not significantly different between healthy and allergic individuals and no clear clustering of the samples was obtained using an unsupervised principal component analysis. However, the analysis of specific bacterial groups allowed us to detect significantly lower levels of bifidobacteria in patients with long-term asthma. Also, in allergic individuals the *Bifidobacterium adolescentis* species prevailed within the bifidobacterial population. The reduction in the levels on bifidobacteria in patients with long-term asthma suggests a new target in allergy research and opens possibilities for the therapeutic modulation of the gut microbiota in this group of patients.

## Introduction

In recent years, growing evidence supporting the “hygiene hypothesis“, which states that a lack of early microbial stimulation results in aberrant immune responses to innocuous antigens later in life, is rising [1,2]. It has also been suggested that modifications of the intestinal microbiota composition that occur as a result of the westernized life-style has disrupted mechanisms that are involved in the development of immunological tolerance [3]. In relation to this, the scientific information about the relationship between allergy and gut microbiota dysbiosis (an imbalance in the gut microbial ecology) is controversial nowadays. While some reports highlight an aberrant microbiota associated with allergic manifestation, such as asthma, rhinitis, and eczema, others did not find any significant differences in the microbial profile, or specific microbial groups, in the gut microbiota of allergic individuals. This could be due to the fact that different allergens can drive allergen-specific responses, to the lack of standardized protocols to analyze the human microbiota, and because the studies were not performed in well-defined population groups. For instance, some studies have indicated an association between the gut microbiota composition and atopic disease, and there is solid evidence that variations of particular intestinal microorganisms might be associated with this physiological condition [4]. Also, a shift of the gut bacterial profiles has been associated with immune disorders in infants and in adults [5–8], as well as in some food allergies, such as milk-hypersensitivity [9,10]. However, despite some positive results, strategies to ameliorate the clinical manifestations of allergy through the modulation of the intestinal microbiota using probiotic microorganisms yielded limited results [11,12].

In this work we performed a cross-sectional study in which we characterized the microbiota of a representative group of adult patients suffering allergic asthma and compared it with a group of healthy controls. We used a 16S rRNA gene-based analysis protocol for this purpose. Significant differences in the bifidobacterial population of asthmatic patients were highlighted.

## Materials and Methods

### Ethical Statement

Ethics approval for this study (reference code AGL2010-14952; grant title “Towards a better understanding of gut microbiota functionality in some immune disorders”) was obtained from the Bioethics Committee of CSIC (Consejo Superior de Investigaciones Científicas) and from the Regional Ethics Committee for Clinical Research (Servicio de Salud del Principado de Asturias) in compliance with the Declaration of Helsinki and we have obtained permission from participants to publish potentially identifying case details. All determinations were performed with fully informed written consent from all participants involved in the study. The study did not interfere with patients’ normal care.

### Study subjects

The study sample comprised 21 patients with allergic asthma (AL codes; 9 male and 12 female; age 39.43 ± 10.98, and 22 healthy controls (HC codes, 7 male and 15 female, age 39.29 ± 9.21). Patient recruitment was carried out in the allergology consultation of the Central University Hospital of Asturias. Information on clinical manifestations was obtained by reviewing clinical records and by personal interviews. Asthma diagnosis was stablished based on the criteria of the Global Initiative for Asthma—GINA [13]. Inclusion criteria also included confirmed diagnosis of asthma (with or without rhinitis) due to perennial allergens. The diagnosis was stablished when patients had a positive skin test and serum-specific IgE levels greater or equal to 3.5 kU/L, with clinically related symptoms. Common relevant antigens were usually house dust mite (HDM), grasses, dog epithelia and a work place allergen (green coffee). All patients were sensitized to HDM and had a positive Skin Prick Test (SPT) result to at least one of the tested allergens.

Serum total IgE, serum-specific IgE testing and SPT were determined at the same time of fecal sampling (Table 1). All patients were diagnosed as persistent asthma with regular control treatment. Most of the patients required daily treatment with inhaled glucocorticosteroid (low or medium dose) alone or with Laba (long-acting beta agonist) (step of treatment 2, 3 or 4 of GINA). Five patients [AL7, AL9, AL11, AL15 and AL16] only required inhaled treatment in a discontinuous way (step 1 or 2 of GINA). All patients excluding AL3, AL7, AL18 and AL19 were suffering perennial rhinitis. AL3, AL7, AL18 and AL19 were suffering intermittent rhinitis or no rhinitis.

**Table 1:** Relevant demographic and clinical features of AL patients.

Sample	Age (years)	Sex	IgE Titer (IU/ml)	SPT	Relevant causal agent*	Specific IgE (KU/l)	Rhinitis duration (years)	Asthma duration (years)
AL1	40	F	160	HDM	*D*. *pteronysinus*	17.5	40	40
AL2	27	F	133	HDM	*D*. *pteronysinus*	32	12	1.5
AL3	24	F	2799	HDM, dog epithelia	Dog epitehlia	>100	-	2.0
AL4	55	M	388	HDM	*D*. *pteronysinus*	16	12	19
AL5	36	M	187	HDM, grasses	*Lepidoglyphus*	28	3	3
AL6	46	F	715	HDM, grasses, green cafe	Green coffe	45	30	20
AL7	38	M	104	HDM	*Lepidoglyphus*	10	-	38
AL8	49	F	173	HDM	*D*. *pteronysinus*	20	10	4
AL9	51	F	389	HDM	Cat epithelia	42	33	3.5
AL10	37	M	235	HDM	*D*. *pteronysinus*	52	32	14
AL11	32	F	608	HDM, grasses	*D*. *pteronysinus*	75	27	15
AL12	54	F	87	HDM	*D*. *pteronysinus*	15	52	5
AL13	38	F	62	HDM	*D*. *pteronysinus*	12	23	11
AL14	57	M	87	HDM	*D*. *pteronysinus*	12	52	32
AL15	27	M	158	HDM	*D*. *pteronysinus*	37	7	23
AL16	37	F	76	HDM	*D*. *pteronysinus*	10	12	4
AL17	50	F	119	HDM	*D*. *pteronysinus*	34	45	11
AL18	22	M	300	HDM	*D*. *pteronysinus*	58	-	3
AL19	22	F	89	HDM	*D*. *pteronysinus*	15	-	7
AL20	42	M	110	HDM	*D*. *pteronysinus*	7	37	34
AL21	44	M	484	HDM	*D*. *pteronysinus*	63	30	38

SPT, Skin Prick Test.

HDM, House Dust Mites.

* More relevant agent in perennial symptoms.

The exclusion criteria considered for this study included subjects diagnosed as having autoimmune diseases, inflammatory bowel disease, or other diseases known to affect intestinal function, as well as subjects who received immunosuppressive therapy and/or allergen specific immunotherapy in the last 5 years (excluding patient AL1 who received an incomplete treatment of immunotherapy [6 months only] 20 months before the fecal sample collection). Patients with oral glucocorticoid or antibiotic treatments during the 6 months prior to the sample collection date were also excluded from the study.

### Skin Prick Tests

Thirteen allergens representing the most relevant inhalant allergens in allergic rhinitis and asthma in Spain according to previous studies [14] (*Dermatophagoides pteronysinus*, *Lepidogliphus destructor*, *Betula berrucosa*, *Cypresus sempervirens*, *Platanus hispánica*, *Lolium perenne* and *Plantago lanceolata*, dog and cat dander, *Alternaria alternata*, *Cladosporium herbarum*, *Peniccillium notatum* and *Aspergillus fumigatus*) were obtained from Leti S.A. (Spain) or Alk Bello (Spain). For skin prick tests, all allergens were standardized using biological units [15]. Green coffee was tested 1/10 weight/volume in saline serum. A SPT was considered positive when the patient developed a wheal larger than a histamine wheal or larger than 3 mm.

### Total IgE and specific IgE determination

InmunoCAP Total IgE and ImmunoCAP Specific IgE (Thermo Fisher) were used to determine the total IgE and specific IgE, respectively. Methods were carried out according to the manufacturer’s instructions.

### Fecal sample collection and DNA extraction

Fresh fecal material (between 10 and 50 grams per person) was collected in sterile containers and immediately manipulated and homogenized within a maximum of 3 hours from defecation. During the time between defecation to homogenization, samples were kept at 4°C. Thirty ml of RNAlater solution (Applied Biosystems, Foster City, CA) were added to 10 grams of sample and the mixture was homogenized in sterile bags, using a stomacher apparatus (IUL Instruments, Barcelona, Spain) (three cycles at high speed, one minute per cycle). Homogenized samples were then stored at -80°C until use. For DNA extraction, samples were thawed and the QIAamp DNA Stool Mini kit was used (Qiagen Ltd., Strasse, Germany), as previously described [16].

### 16S rRNA gene amplification and sequencing of 16S rRNA gene-based amplicons

Primer selection, amplicon generation and purification, amplicon library construction and sequencing were performed essentially as described by Hevia et al. [17]. Briefly, the primer pair Probio_Uni / Probio_Rev [16] was used to generate amplicon pools of approximately 200 bp length. The integrity of the PCR amplicons was analyzed by electrophoresis prior to their purification using the Wizard SV Gen PCR Clean-Up System (Promega, Madison, WI), and the Agencourt AMPure XP DNA purification beads (Beckman Coulter Genomics GmbH, Bernried, Germany). Libraries for each run were diluted to 3E9 DNA molecules prior to clonal amplification. Emulsion PCR was carried out using the Ion OneTouch^™^ 200 Template Kit v2 DL (Life Technologies, Guilford, CA) and sequencing of the amplicon libraries was carried out on 316 chips using the Ion Torrent PGM system and employing the Ion Sequencing 200 kit (Life Technologies). After sequencing, individual sequence reads were filtered by the PGM software to remove low quality and polyclonal sequences. Sequences matching the PGM 3’ adaptor were also automatically trimmed. All PGM quality-approved, trimmed and filtered data were exported as SFF files.

### Sequence-based microbiota analysis

The SFF files were processed using QIIME [18]. Quality control retained sequences with an average length of 180 bp (S1 Table), mean sequence quality score >25, with truncation of a sequence at the first base if a low quality rolling 10 bp window was found. Presence of homopolymers >7 bp, and sequences with mismatched primers were omitted. In order to calculate alpha and beta diversity indices, 16S rRNA Operational Taxonomic Units (OTUs) were defined at ≥ 97% sequence homology using uclust [19]. Chimeric sequences were removed using ChimeraSlayer [20]. All reads were classified to the lowest possible taxonomic rank using QIIME and a reference dataset from the Ribosomal Database Project [21].

### Nucleotide sequence accession number

The raw sequences reported in this article have been deposited in the NCBI Short Read Archive (SRA) (study accession number: PRJNA276631).

### Statistical analyses

Statistical analysis was performed using IBM-SPSS version 19.0 (IBM SPSS, Inc., Chicago, IL). Individuals were ordered according to their sequence data composition by Principal Component Analysis using the taxonomic data at the phylum and family level. Patterns were extracted using all the variation from the taxonomic data using an indirect method as model, and allergy as metadata. Significant differences between healthy controls and asthmatic patients were only considered below a p-value of 0.05. The p-values at the phylum, family and genus level are included in S2 Table. Multivariate linear regression was performed in order to identify the clinical and demographical features associated with the levels of *B*. *adolescentis* in asthmatic individuals. The statistical parameters employed were β (standardized regression coefficient) and R^2^ (coefficient of multiple determinations). The conventional probability value for significance (0.05) was used in the interpretation of results.

## Results

**Fig 1:**
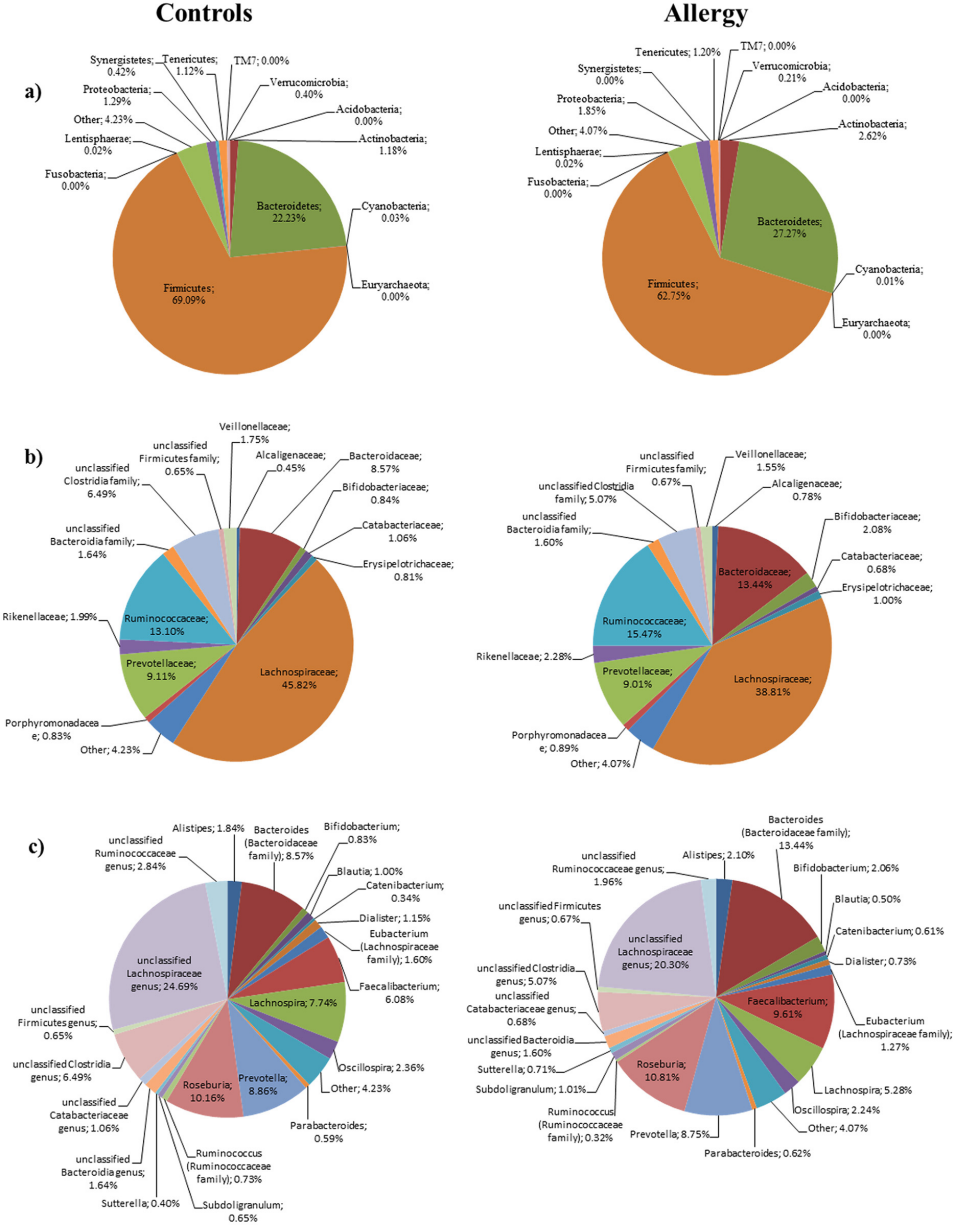
Aggregate microbiota composition in faecal samples from healthy controls and allergic asthma patients at phylum level (panel a), family level (panel b) and genus level (panel c). In panels b and c only taxonomic groups above 0.5% are shown.

**Fig 2:**
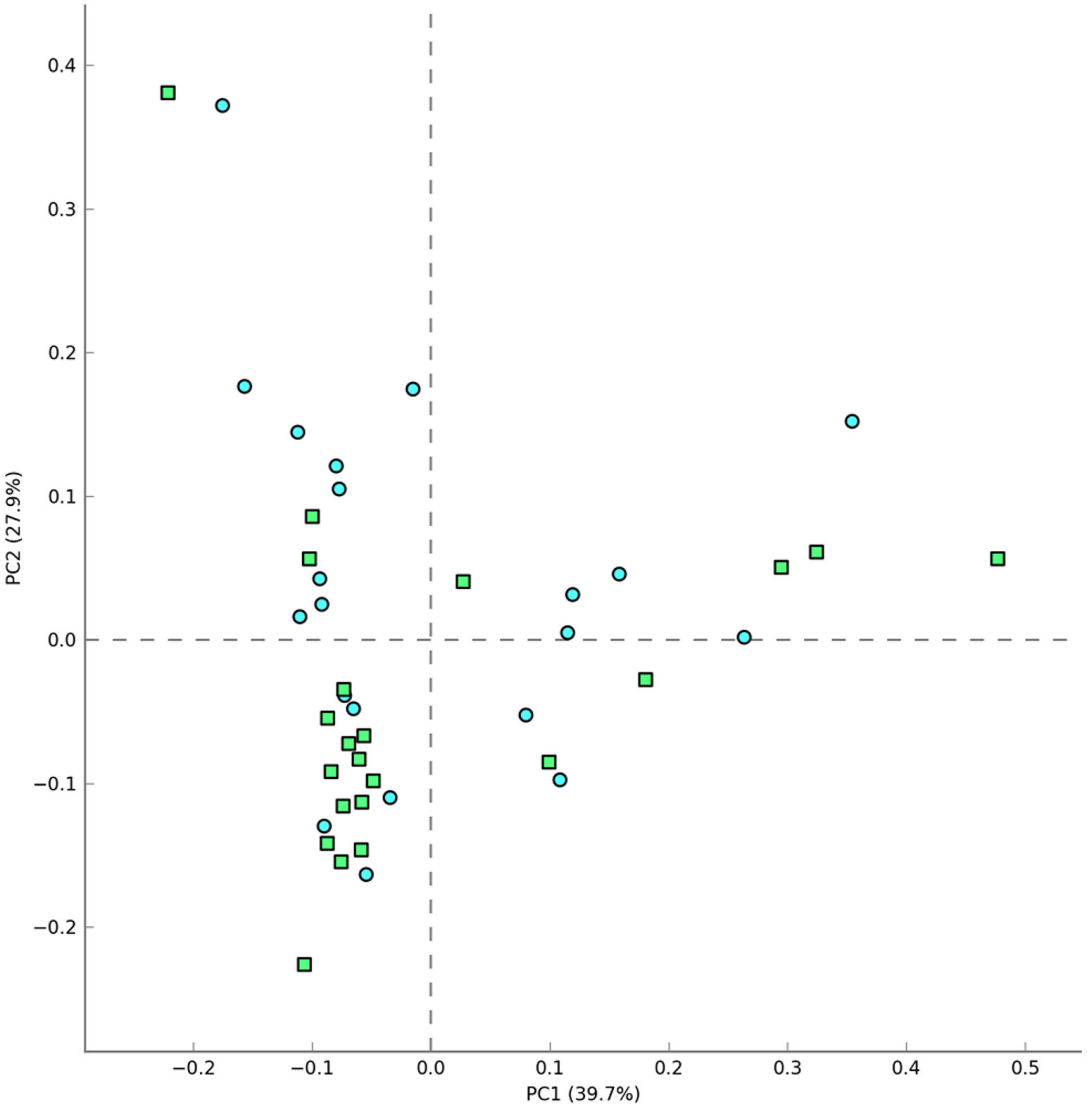
Principal Component Analysis using the 16S rRNA metagenomic profiles and the genus level. Presence/absence of asthma was further included as metadata. (Green squares: healthy controls; blue circles: asthmatic patients).

To shed some light on the potential relationship between the human microbiota and allergy, we analyzed the fecal microbiota of 21 subjects suffering from allergic asthma. We followed strict exclusion criteria trying to minimize the influence of environmental and genetic factors on our analysis. We considered a homogeneous group, with a very specific pathology: confirmed allergic asthma. Clinical history of the patients and the evidence of an IgE mediated disease are the main criteria in our analysis, in order to associate the microbiota profile in a highly selected allergic group. Fig 1 shows the microbiota composition at phylum, family and genus level that was obtained for healthy individuals and asthmatic persons. Rarefaction curves obtained by plotting the Shannon and Chao1 indexes against the number of sequences (S1 Fig) showed that a large part of the diversity of the samples was detected. We did not obtain significant alpha-diversity indexes (Chao1, PD Whole Tree, Observed Species, Shannon and Simpson indexes) between asthmatic and healthy individuals (data not shown). Furthermore, individuals were ordered according to their sequence data composition by Unsupervised Principal Component Analysis (PCA) using the taxonomic data at the phylum, family and genus level. No clear clustering of the samples was obtained at the three different levels analyzed (Fig 2 shows the plot obtained using genus level), suggesting that the characteristics of the microbiota are similar considering the sample sets of sequences in both population groups.

**Fig 3:**
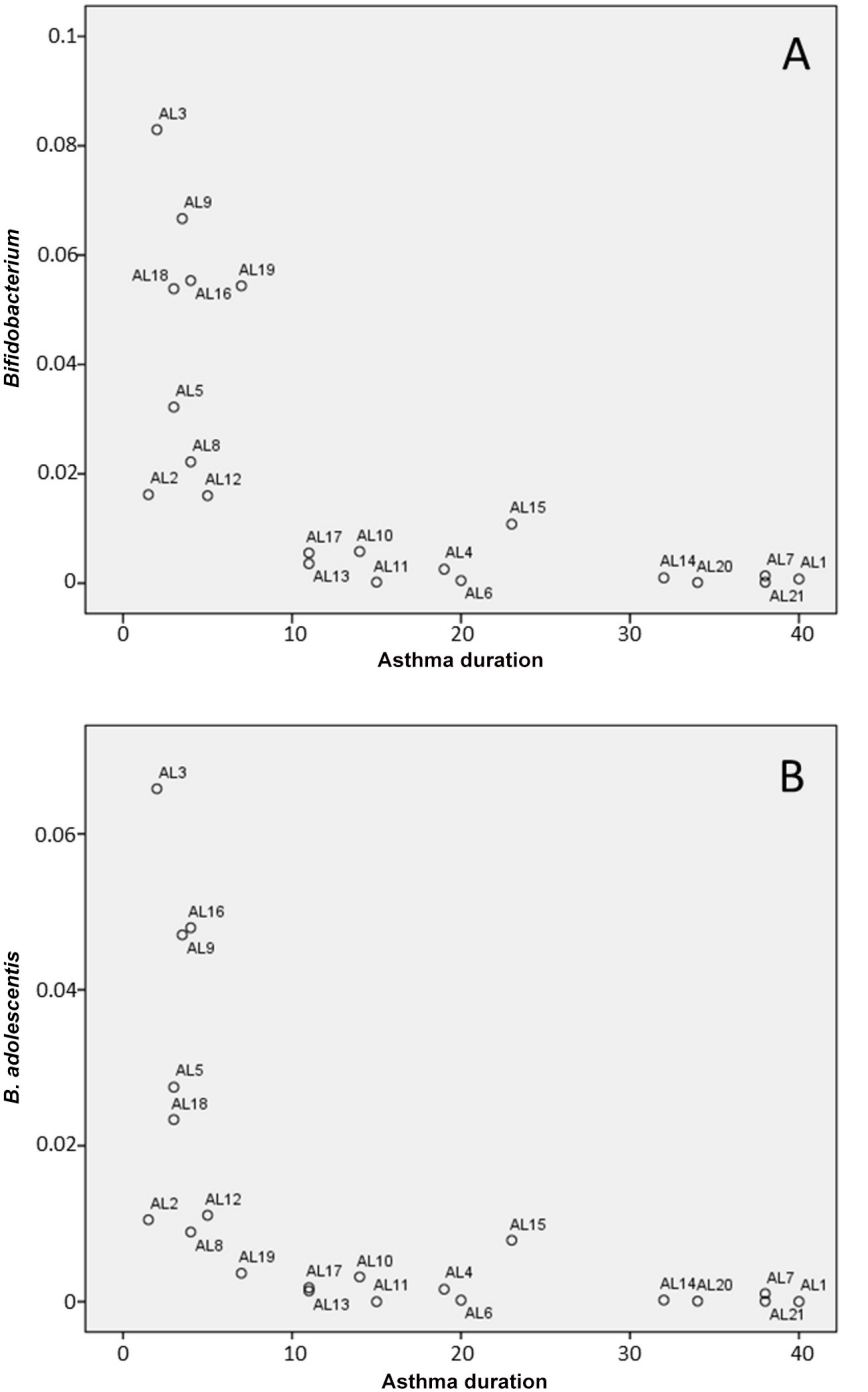
Correlation between the time of asthma ailment and the abundance of bifidobacteria (A) and *B*. *adolescentis* (B) in allergic asthma patients.

In our study, we organized relevant data of the patients (age, sex, total and specific IgE titers, causal agent, rhinitis, disease duration) in a metadata file for all the microbiota profiles. Both metadata/microbiota profiles were loaded in STAMP v2.0.3 and a PCA analysis performed using the variability of the 16S rRNA gene profiling at different taxonomic levels. Sample classification according to the metadata revealed no specific clustering of the samples or correlation with different clinical features and analytical results. Furthermore, our results do not stablish a link between microbial groups and asthma when the fecal microbiota was analyzed in its entirety, and statistically significant differences between healthy controls and asthmatic patients were only detected for *Faecalibacterium* and *Bifidobacterium* at the genus level (both genera were more abundant in allergic asthma patients; S2 Table). However, when we examined the possible relationship between the bacterial groups present in the fecal samples and the time suffering from asthma, we found that low levels of bifidobacteria correlate with long ailment periods of asthma (Fig 3). Although the amplicons of the 16S sequences could be relatively short to perform a totally reliable population structure analysis at species level, it is noteworthy that the relative abundance of sequences assigned to *Bifidobacterium adolescentis* was significantly higher in allergic asthma samples. Remarkably, in asthmatic individuals the *B*. *adolescentis* species prevailed, but in healthy controls a group of other species (*Bifidobacterium longum*, *Bifidobacterium breve* and *Bifidobacterium bifidum*) constituted the majority of the bifidobacterial population. Furthermore, if we subcategorize the allergic asthma patients considering the median for asthma ailment (11 years), the means of the *B*. *adolescentis* population in the two groups (long-term asthma and short-term asthma groups; > or < 11 years suffering from asthma) are significantly different (ANOVA test, p<0.002). Remarkably, our observations were significantly relevant for allergic asthma but not for duration of allergic rhinitis. Furthermore, using a Spearman Ranked Scores analysis, the levels of *Bifidobacterium* and of *B*. *adolescentis* were negatively correlated with the asthma duration (correlation coefficient -0,84074, p-value: 3,4588E-06 for *Bifidobacterium*; correlation coefficient -0,84682, p-value: 2,494E-06 for *B*. *adolescentis*). On the other hand, a multivariate linear analysis showed that the time suffering the disease and the IgE titer were identified as *B*. *adolescentis* predictor variables. We found that there is a negative association of *B*. *adolescentis* with the time suffering the disease (β = -0.457), and a positive association with total IgE levels (β = 0.444), p model <0.001. Other clinical or demographical variables such as sex, age, most relevant causal allergen, specific IgE or time of rhinitis disease did not show any association.

## Discussion

The microbiota plays an integral role in the homeostasis of multiple interconnected host metabolic and immune networks. There is solid experimental evidence that gut microbiota regulates our inflammatory immune response [22]. Disturbed gut colonization patterns have been associated with allergic disease but whether microbial variation is the cause or effect of these diseases is still under investigation. Whereas some authors have established a link between specific microbial profiles and allergy, other authors suggest that this should not be considered in broad terms and that it depends on the clinical manifestation of the allergic reaction and of the specific antigens triggering the immune response [9, 23,24].

Previous studies in infant/children populations have shown that lower levels of bifidobacteria are found in feces of allergic subjects compared with non-allergic individuals. However, the present work, performed in a well-defined population of allergic asthma adults, shows that the relative abundance of bifidobacteria is higher in the asthma group, suggesting an influence of this particular pathology on shaping bifidobacterial ecology in the gut. In relation to this, infants of atopic mothers were the only ones to be colonized with *B*. *adolescentis* and infants from allergic mothers had lower counts of bifidobacteria in feces than infants from non-allergic mothers [25]. Furthermore, Stsepetova et al. [26] indicated a less diverse composition of intestinal microbiota and prevalence of *B*. *adolescentis* in allergic childrens, and He et al. [27] found that *B*. *adolescentis* is found more often in the fecal microbiota of allergic childrens than in non-allergic ones. Thus, from previous works it seems that there is an association between *B*. *adolescentis* and allergy. The novelty of our findings is that, to our knowledge, this is the first time that bifidobacteria in general, and *B*. *adolescentis* in particular, has been associated with the ailment of allergic asthma. Remarkably, higher levels of *Faecalibacterium* were detected in the asthmatic patients; this finding could be related to the regular treatment with anti-inflammatory drugs of most of the patients, since the presence of *Faecalibacterium* is normally decreased during inflammatory process [28].

In summary, our data suggest that there is not a significant difference between the fecal microbiota profile of allergic subjects suffering from asthma and healthy individuals, neither a different microbial diversity. However, lower bifidobacterial levels are present in asthmatic individuals with long-term asthma, compared with those whose asthma was diagnosed more recently. Our findings suggest that the differences in the bifidobacterial populations in control vs asthma groups, as well as in short-term vs long-term asthma groups, could be explored further in order to develop novel treatments based on gut microbiota modulation. The results obtained also contribute to generate knowledge on the potential role of bifidobacteria in the physiology of allergy, and support the potential use of strains of these genera as probiotics to modulate allergy symptoms.

## Supporting Information

S1 Fig Rarefaction curves generated for 16S rRNA gene sequences obtained from faecal samples of control subjects (HC samples) and allergic asthma patients (AL samples). Panel a represents the rarefaction curves using the Shannon index. Panel b displays rarefaction curves using the Chao1 index.
(DOCX)

S1 Table Sequence data features. (DOCX)

S2 Table Statistically significant differences between healthy controls (HC) and patients (AL) at the phylum, family and genus levels. (XLSX)
