# Supplementary figures and images for: Allergic Patients with Long-Term Asthma Display Low Levels of Bifidobacterium adolescentis
Source: PLoS One. 2016 Feb 3;11(2):e0147809. doi: 10.1371/journal.pone.0147809 (PMC4739579; doi:10.1371/journal.pone.0147809)

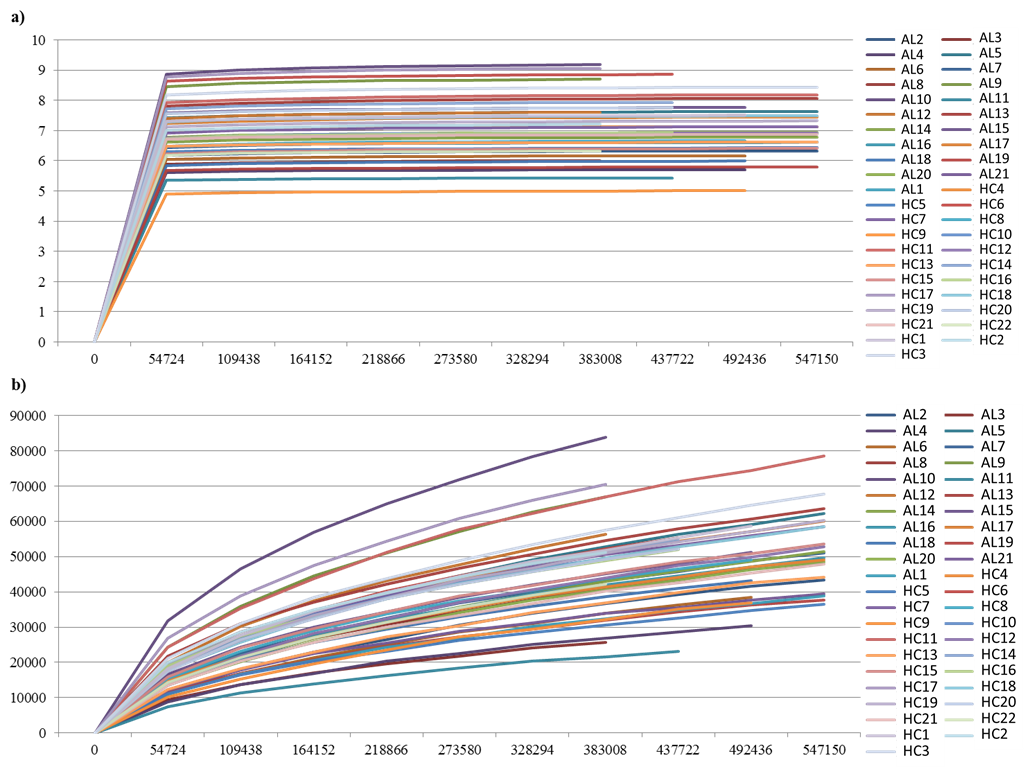

Supplement: S1 Fig — Panel a represents the rarefaction curves using the Shannon index. Panel b displays rarefaction curves using the Chao1 index. (DOCX) [file pone.0147809.s001.docx]
