# Supplementary material for: Allergic Patients with Long-Term Asthma Display Low Levels of Bifidobacterium adolescentis
Source: PLoS One. 2016 Feb 3;11(2):e0147809. doi: 10.1371/journal.pone.0147809 (PMC4739579; doi:10.1371/journal.pone.0147809)
Supplement: S1 Table — (DOCX) [file pone.0147809.s002.docx]

| **Dataset** | **Sample** | **Number of reads** | **Number of reads removed because of:** | | | | | | **Final reads number** | **Reduced by (%)** | **average reads length** | **Sequence Output (Mbp)** |
| --- | --- | --- | --- | --- | --- | --- | --- | --- | --- | --- | --- | --- |
|  |  |  | **Outside bounds (150-400)** | **Ambiguous bases** | **Mean quality <25** | **Homopolymer runs >7bp** | **Primer mismatch >1** | **Low quality windows truncation results in <150bp** |  |  |  |  |
| **Healthy controls** | HC1 | 705107 | 117184 | 0 | 0 | 485 | 49256 | 22309 | 515873 | 26.84% | 187.2 | 96.57 |
|  | HC2 | 516755 | 64323 | 0 | 0 | 701 | 30268 | 16062 | 405401 | 21.55% | 189.1 | 76.66 |
|  | HC3 | 800844 | 154617 | 0 | 0 | 1175 | 49787 | 35075 | 560190 | 30.05% | 186.3 | 104.36 |
|  | HC4 | 750409 | 102522 | 0 | 0 | 646 | 27941 | 24678 | 594622 | 20.76% | 192.2 | 114.29 |
|  | HC5 | 2043202 | 542937 | 0 | 0 | 626 | 81256 | 59920 | 1358463 | 33.51% | 196.5 | 266.94 |
|  | HC6 | 682959 | 157189 | 0 | 0 | 7249 | 19937 | 38886 | 459698 | 32.69% | 191.1 | 87.85 |
|  | HC7 | 948930 | 145829 | 0 | 0 | 1367 | 60152 | 26943 | 714639 | 24.69% | 187.1 | 133.71 |
|  | HC8 | 624186 | 91195 | 0 | 0 | 705 | 20792 | 15731 | 495763 | 20.57% | 186.4 | 92.41 |
|  | HC9 | 651188 | 80640 | 0 | 0 | 498 | 28704 | 13714 | 527632 | 18.97% | 196.7 | 103.79 |
|  | HC10 | 588180 | 158159 | 0 | 0 | 483 | 21507 | 22471 | 385560 | 34.45% | 186.5 | 71.91 |
|  | HC11 | 1324073 | 418738 | 0 | 0 | 2426 | 35519 | 33300 | 834090 | 37.01% | 187.4 | 156.31 |
|  | HC12 | 822090 | 99350 | 0 | 0 | 855 | 59218 | 19277 | 643390 | 21.74% | 189.3 | 121.79 |
|  | HC13 | 1260689 | 160710 | 0 | 0 | 5575 | 87280 | 29647 | 977477 | 22.46% | 196.3 | 191.88 |
|  | HC14 | 592338 | 73766 | 0 | 0 | 2497 | 34182 | 19793 | 462100 | 21.99% | 383.9 | 177.40 |
|  | HC15 | 842264 | 225177 | 0 | 0 | 197 | 28266 | 13183 | 575441 | 31.68% | 187.6 | 107.95 |
|  | HC16 | 647372 | 154647 | 0 | 0 | 672 | 23038 | 23939 | 445076 | 31.25% | 186.3 | 82.92 |
|  | HC17 | 766684 | 297828 | 0 | 0 | 2071 | 16239 | 40393 | 410153 | 46.50% | 184 | 75.47 |
|  | HC18 | 803461 | 169323 | 0 | 0 | 4049 | 30558 | 24060 | 575471 | 28.38% | 189.6 | 109.11 |
|  | HC19 | 1419470 | 189686 | 0 | 0 | 1954 | 90892 | 38689 | 1098249 | 22.63% | 188.6 | 207.13 |
|  | HC20 | 638109 | 145072 | 0 | 0 | 777 | 21445 | 17367 | 453448 | 28.94% | 192 | 87.06 |
|  | HC21 | 977825 | 148439 | 0 | 0 | 1041 | 56972 | 28454 | 742918 | 24.02% | 196 | 145.61 |
|  | HC22 | 591244 | 140515 | 0 | 0 | 567 | 36224 | 15913 | 398025 | 32.68% | 189.7 | 75.51 |
| **Allergic patients** | AL1 | 1572245 | 139941 | 0 | 0 | 5167 | 52450 | 32456 | 1342231 | 14.63% | 193.7 | 259.99 |
|  | AL2 | 1059336 | 130980 | 0 | 0 | 1867 | 49227 | 17949 | 859313 | 18.88% | 193.1 | 165.93 |
|  | AL3 | 664346 | 236268 | 0 | 0 | 97 | 17048 | 7916 | 403017 | 39.34% | 197.4 | 79.56 |
|  | AL4 | 653445 | 75093 | 0 | 0 | 196 | 50862 | 8821 | 518473 | 20.66% | 193.8 | 100.48 |
|  | AL5 | 872568 | 119802 | 0 | 0 | 2277 | 51997 | 29505 | 668987 | 23.33% | 191.3 | 127.98 |
|  | AL6 | 671449 | 93278 | 0 | 0 | 580 | 38027 | 9885 | 529679 | 21.11% | 190.9 | 101.12 |
|  | AL7 | 808550 | 87389 | 0 | 0 | 1207 | 54105 | 20770 | 645079 | 20.22% | 193.6 | 124.89 |
|  | AL8 | 709243 | 115210 | 0 | 0 | 1048 | 27934 | 18376 | 546675 | 22.92% | 384 | 209.92 |
|  | AL9 | 714325 | 253623 | 0 | 0 | 303 | 21664 | 30939 | 407796 | 42.91% | 190 | 77.48 |
|  | AL10 | 692806 | 202082 | 0 | 0 | 1463 | 18656 | 52644 | 417960 | 39.67% | 184 | 76.90 |
|  | AL11 | 610199 | 68204 | 0 | 0 | 184 | 44709 | 7942 | 489160 | 19.84% | 197.7 | 96.71 |
|  | AL12 | 537164 | 89365 | 0 | 0 | 1594 | 23642 | 22409 | 400154 | 25.51% | 192.2 | 76.91 |
|  | AL13 | 811040 | 157438 | 0 | 0 | 1821 | 63140 | 41485 | 547156 | 32.54% | 186.9 | 102.26 |
|  | AL14 | 725363 | 99333 | 0 | 0 | 1073 | 20764 | 17652 | 586541 | 19.14% | 377.1 | 221.18 |
|  | AL15 | 658803 | 84063 | 0 | 0 | 1157 | 41492 | 28947 | 503144 | 23.63% | 190.9 | 96.05 |
|  | AL16 | 1113622 | 274500 | 0 | 0 | 3057 | 36169 | 27199 | 772697 | 30.61% | 190.4 | 147.12 |
|  | AL17 | 1363487 | 283794 | 0 | 0 | 5947 | 43480 | 58481 | 971785 | 28.73% | 189 | 183.67 |
|  | AL18 | 667831 | 78075 | 0 | 0 | 535 | 47619 | 15217 | 526385 | 21.18% | 188.2 | 99.07 |
|  | AL19 | 1115415 | 159116 | 0 | 0 | 1370 | 51199 | 24859 | 878871 | 21.21% | 191.1 | 167.95 |
|  | AL20 | 926394 | 158659 | 0 | 0 | 1045 | 30000 | 14130 | 722560 | 22.00% | 189.3 | 136.78 |
|  | AL21 | 840647 | 162457 | 0 | 0 | 227 | 31866 | 16731 | 629366 | 25.13% | 195.3 | 122.92 |
